# Poster Session I - A178 BARRIERS TO TIMELY DIAGNOSIS OF EARLY-ONSET COLORECTAL CANCER: A SYSTEMATIC REVIEW AND QUALITATIVE ANALYSIS

**DOI:** 10.1093/jcag/gwaf042.178

**Published:** 2026-02-13

**Authors:** B Nguyen, J Lim

**Affiliations:** Medicine, The University of British Columbia Faculty of Medicine, Vancouver, BC, Canada; Medicine, The University of British Columbia Faculty of Medicine, Vancouver, BC, Canada

## Abstract

NOT PUBLISHED AT AUTHOR’S REQUEST

Please acknowledge all funding agencies listed below

**Funding Agencies:**

None

